# Impact of Chinese criteria on potentially inappropriate medication use in China

**DOI:** 10.7189/jogh.15.04063

**Published:** 2025-02-07

**Authors:** Fangyuan Tian, Zhaoyan Chen, Ying Zhang, Qiyi Feng, Xi Chen

**Affiliations:** 1 Department of Pharmacy, National Clinical Research Center for Geriatrics, West China Hospital, Sichuan University, Chengdu, China; 2 Department of Epidemiology and Health Statistics, West China School of Public Health and West China Fourth Hospital, Sichuan University, Chengdu, China; 3 Precision Medicine Research Center, Sichuan Provincial Key Laboratory of Precision Medicine and National Clinical Research Center for Geriatrics, West China Hospital, Sichuan University, Chengdu, China; 4 Department of Integrated Care Management Center, West China Hospital, Sichuan University, Chengdu, China

## Abstract

**Background:**

In 2018, China issued a set of criteria for effectively identifying and managing potentially inappropriate medication (PIM) use in older adults. However, there is currently a lack of evidence regarding the impact of these criteria on PIM use among older Chinese adults.

**Methods:**

We used interrupted time series analysis on the prescription data of older outpatients from 59 hospitals in six major geographic regions of China to compare changes in the overall prevalence of PIM use, the prevalence of PIM use stratified by different numbers of PIMs, and the prevalence of top five PIMs (*i.e.* clopidogrel, estazolam, zolpidem, sliding-scale insulin, and alprazolam) from 2015 (before) to 2021 (after) the release of criteria in 2018.

**Results:**

We included 982 605 older outpatients. Compared with trends prior to the publication of the criteria, there were significant decreases in the coefficient for change in the slope of the overall prevalence of PIM use (β = −0.607; 95% confidence interval (CI) = −0.881, −0.482; *P* < 0.001), the prevalence of single PIM use (β = −0.368; 95% CI = −0.465, −0.272; *P* < 0.001), the prevalence of multiple PIM use (β = −0.104; 95% CI = −0.173, −0.080; *P* = 0.019), the prevalence of clopidogrel (β = −0.342; 95% CI = −0.463, −0.227; *P* = 0.006), and the prevalence of estazolam (β = −0.077; 95% CI = −0.124, −0.037; *P* = 0.009) post-publication. Conversely, there was a significant increase in the prevalence of zolpidem, after the criteria were released (β = 0.030; 95% CI = 0.002, 0.057; *P* = 0.036).

**Conclusions:**

We found that the release of criteria for effectively identifying and managing PIM use has had a positive effect on its prevalence among older outpatients in China.

Global life expectancy is projected to increase from 73.6 years in 2022 to 78.2 years by 2050, indicating a deepening global ageing trend [1]. China is home to a fifth of the world’s older people [2], and the results of its seventh national population census revealed that in the past ten years, the share of the population aged >65 years has increased by 4.6% (*i.e.* from 8.9 to 13.5%) [3]. The burden of chronic diseases has also significantly increased with the continuous growth of the ageing population, and older adults often must use multiple medications [4]. As the number of drugs used by older adults increases, along with changes in pharmacokinetics and pharmacodynamics, the risk of drug-drug interactions and adverse drug reactions (ADRs) in certain disease states also increases.

The safety of medication in older adults is a critical issue that warrants greater attention. When the potential adverse risks of a medication used by older adults exceed the expected benefits, this is classified as a potentially inappropriate medication (PIM) [5,6]. Currently, PIM is a significant factor contributing to increased medication safety risks in older adults [7], ranking as the third most common cause of hospital admissions and the leading cause of hospital-acquired conditions among older adults [8]. Studies have shown that older adults using PIM have greater risks of mortality, osteoporosis, falls, and readmission than not using them [9–12], which severely impacts their quality of life [13].

To facilitate the identification and management of PIM use, various countries have developed criteria for PIM [14]. Among the most widely used criteria are the Beers and the Screening Tool of Older Person's Prescriptions/Screening Tool to Alert Doctors to the Right (STOPP/START) criteria [15], based on which the Chinese criteria were developed by incorporating data from three national ADR monitoring centres and reports from 22 hospitals in Beijing. The criteria were further refined via the Delphi method based on expert opinions and officially published in 2018 as the national criteria [16]. A recent analysis found that the global pattern in the prevalence of PIM use among older adults in outpatients was 36.7%, and that PIM use among older adults has become increasingly prevalent in the past two decades [17]. In China, older adults with chronic conditions primarily receive their medications through outpatient services, where the issue of PIM use is particularly prevalent [18].

One study analysed 15 932 older patients in Canada from 2011–19 to explore the impact of discharge medication review interventions on the prevalence of PIM use [19]. These interventions, implemented in 2015, resulted in a downward trend in the prevalence of PIM use in older inpatients discharged from tertiary hospitals. Similarly, 50 924 prescriptions from eight older outpatient services in the USA were analysed from 2000–04 to explore the impact of computerised decision support systems on PIM use [20]. The results revealed a decrease in the prevalence of PIM use following the implementation of these systems. In our view, the release of the PIM criteria will also play a positive role in the management of PIM use. Therefore, we aimed to explore the changes in the prevalence of PIM use among older outpatients before and after the release of the criteria for PIM. As this is a criterion applied nationwide, the external validity of our findings may be stronger compared to other similar studies.

Understanding the effect of the criteria on the prevalence of PIM use among older adults can help assess the effectiveness and outcomes of its implementation, providing valuable insights for government agencies and policymakers to improve health care services. We referred to the impact of the release of the Chinese criteria as reductions in PIM use among older outpatients via interrupted time series analysis. Our findings could therefore provide evidence for the prevention, intervention, and management of PIM use in older adults.

## METHODS

### Study population, setting, and data source

We obtained the data from a hospital prescription analysis cooperation project led by the Chinese Pharmaceutical Association (CPA), which included six cities (Beijing Shanghai, Guangzhou, Shenyang, Zhengzhou, and Chengdu) representing the six major geographic regions of China (North China, East China, South China, Northeast China, Central China, and Southwest China). The CPA research team used purposive sampling to select several secondary-level and tertiary-level hospitals in each city, identifying them based on their clinical experience. For older outpatients at these institutions, the CPA research team took a random sample quarterly over ten days (split into two five-day periods). This can ensure that the extracted data are evenly distributed throughout the year to avoid the seasonality of a single period affecting the representativeness of the overall results. Quarterly sampling can better support the analysis of time trends and facilitate the observation of the effect of long-term changes or breakpoints.

We included prescriptions for older (aged ≥65 years) outpatients and excluded patients if they did not receive any medications and were only prescribed items such as sterile water for injection or contrast agents. We adhered to the STROBE guidelines in reporting our findings [21].

### Diagnosis and medication classification

The CPA research team classified and organised prescription diagnoses based on the International Classification of Diseases, 10th Revision, and drug categories according to the World Health Organization's Anatomical Therapeutic Chemical classification system and generic drug names. For prescriptions missing clinical diagnoses, the research team contacted the relevant medical institutions by phone for clarification.

### Evaluation criteria

We used the Chinese criteria to assess PIM use in older adults. The evaluation process involved two clinical pharmacists (ZC, YZ) specialising in chronic diseases who independently conducted assessments via an information system. After completing their assessments, a clinical pharmacist specialising in geriatrics (FT) manually reviewed and verified the results. Any discrepancies between the initial assessments were resolved through discussion among the three researchers.

### Study periods

The prescription period spanned from 1 January 2015, to 31 December 2021. The criteria were released in February 2018. Therefore, the study includes data from two distinct periods:13 quarters before the release (the first quarter of 2015 to the first quarter of 2018) and 15 quarters after the release (the second quarter of 2018 to the fourth quarter of 2021).

### Outcomes

Our outcomes included the overall prevalence of PIM use, the prevalence of PIM use stratified by the number of PIMs, and the prevalence of PIM use linked to the top five PIMs, which we chose based on frequency. We chose the number of PIMs as an outcome measure because the complexity of clinical interventions increases with the number of PIMs in a prescription. We selected the top five PIMs because they account for the most detected PIMs, and because the implementation of control measures for these top five PIMs can significantly increase management efficiency and reduce the overall prevalence of PIM use.

### Statistical analysis

We used interrupted time series analysis [22] to compare changes in outcomes before and after the release of the criteria. We defined our expected impact model as both the change in level and the change in slope. The coefficient β_0_ represented the constant term, indicating the initial level of the outcome variable. The coefficient β_1_ corresponded to the slope of the prevalence of PIM use before the release of the criteria. The coefficient β_2_ represented the change in the level of the prevalence of PIM use after the release of the criteria, and the coefficient β_3_ the change in the slope of the prevalence of PIM use following the release of the criteria.

We used the Durbin-Watson (DW) statistics to assess the presence of first-order autocorrelation. This statistics ranges between 0–4, with values close to 2 indicating no autocorrelation (*P* ≥ 0.05). If the value deviates significantly from 2, indicating the presence of first-order autocorrelation (*P* < 0.05), the generalised least squares method is applied, implemented through the Prais-Winsten statistics [23]. We conducted all analyses in *R*, version 4.2.0 (R Core Team, Vienna, Austria) and considered a two-sided *P* < 0.05 as statistically significant.

### Ethical approval

The Sichuan University West China Hospital Research Ethics Board (2024/810) provided ethical approval for our study. No individual patient consent was needed, as all the data were deidentified.

## RESULTS

### Patients characteristics

We analysed data for 982 605 outpatients from 59 hospitals, spanning the period from 2015 to 2021 (Table S1 in the **Online Supplementary Document**). There were 291 964 PIMs in total, and the prevalence of PIM use was 29.71%. The number of outpatients and PIM increased from 2015 to a peak in 2019, before declining in 2021 (Tables S2 and S3 in the **Online Supplementary Document**). Among the six cities, Shanghai had the highest number of patients and PIM, while Zhengzhou had the lowest (**Table 1**). Secondary-level hospitals accounted for 83 515 patients (8.50%) and 23 666 PIMs (8.11%), while tertiary-level hospitals accounted for the majority of our sample in both cases, with 899 090 patients (91.50%) and 268 298 PIMs (91.89%). Male patients received 627 329 prescriptions (63.84%) and 190 619 PIMs (65.29%), whereas female patients received 355 276 prescriptions (36.16%) and 101 345 PIMs (34.71%). The median age in our sample was 81 (IQR = 73–87) years, with the oldest older patients (≥80 years) receiving prescriptions accounting for 56.10%.

**Table 1:** Basic characteristics of patients*

	Total	PIM group	Non-PIM group	*P*-value
**Total number of participants**	982 605	291 964 (29.71)	690 641 (70.29)	
**City**				<0.001
Beijing	203 342 (20.69)	72 028 (24.67)	131 314 (19.01)	
Chengdu	80 940 (8.24)	29 229 (10.01)	51 711 (7.49)	
Guangzhou	140 311 (14.28)	54 525 (18.68)	85 786 (12.42)	
Shanghai	324 229 (33.00)	80 199 (27.47)	244 030 (35.33)	
Shenyang	214 352 (21.81)	47 911 (16.41)	166 441 (24.10)	
Zhengzhou	19 431 (1.98)	8072 (2.76)	11 359 (1.64)	
**Hospital level**				<0.001
Secondary	83 515 (8.50)	23 666 (8.11)	59 849 (8.67)	
Tertiary	899 090 (91.50)	268 298 (91.89)	630 792 (91.33)	
**Sex**				<0.001
Male	627 329 (63.84)	190 619 (65.29)	436 710 (63.23)	
Female	355 276 (36.16)	101 345 (34.71)	253 931 (36.77)	
**Age in years, MD (IQR)**	81 (73–87)			<0.001
**Age in years, stratified by age groups**				<0.001
65–79	431 372 (43.90)	112 471 (38.52)	318 901 (46.17)	
≥80	551 233 (56.10)	179 493 (61.48)	371 740 (53.83)	

IQR – interquartile range, MD – median, PIM – potentially inappropriate medication

*Data are presented as n (%) unless specified otherwise.

### PIM use in older outpatients

Among the 291 964 patients with PIM use, 236 688 (81.07%) had at least one PIM and 55 276 had multiple PIMs (**Table 2**), accounting for for 373 018 PIMs in total. The top five medications of PIM use recognised by the criteria were estazolam, sliding-scale insulin, zolpidem, and alprazolam, accounting for 56.26% of all PIMs.

**Table 2:** PIM use among patients*

	2015	2016	2017	2018	2019	2020	2021	Total
**Total number of patients**	116 037	138 669	143 232	154 141	165 481	133 151	131 894	982 605
**Patients with PIM**	32 599	41 752	44 009	48 394	48 625	38 340	38 245	291 964
**Single PIM**	27 143 (74.06)	34 581 (82.82)	35 782 (81.31)	38 892 (80.37)	39 254 (80.73)	30 817 (80.38)	30 219 (79.01)	236 688 (81.07)
**Multiple PIMs**	5456 (16.74)	7171 (17.18)	8227 (18.69)	9802 (19.63)	9371 (19.27)	7523 (19.62)	8026 (20.99)	55 276 (18.93)
**PIM**	42 560	51 064	54 692	60 797	64 149	50 362	49 394	373 018
**Clopidogrel**	9795 (8.44)	13 991 (10.09)	14 130 (9.87)	14 248 (9.24)	13 873 (8.38)	9252 (6.95)	8893 (6.74)	84 182 (8.57)
**Estazolam**	4724 (4.07)	6491 (4.68)	6993 (4.88)	7792 (5.06)	8127 (4.91)	7105 (5.34)	6643 (5.04)	47 875 (4.87)
**Sliding-scale insulin**	3998 (1.64)	4583 (1.71)	4775 (2.04)	5115 (2.40)	5541 (2.77)	4208 (2.90)	4253 (3.42)	32 473 (3.30)
**Zolpidem**	1899 (3.45)	2373 (3.30)	2927 (3.33)	3692 (3.32)	4586 (3.35)	3868 (3.16)	4510 (3.22)	23 855 (2.43)
**Alprazolam**	1988 (1.71)	2377 (1.71)	2898 (2.02)	3444 (2.23)	3597 (2.17)	3557 (2.67)	3606 (2.73)	21 467 (2.18)
**Total**	22 404	29 815	31 723	34 291	35 724	27 990	27 905	209 852

PIM – potentially inappropriate medication

*Data are presented as n (%) unless specified otherwise.

### Trend changes in the prevalence of PIM use

**Figure 1:**
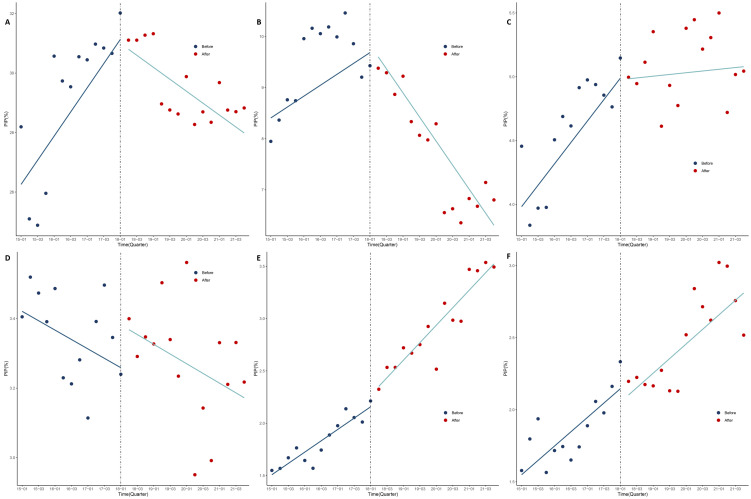
Prevalence of PIM use with top five PIM before and after the criteria. **Panel A.** Overall prevalence of PIM use. **Panel B.** Clopidogrel. **Panel C.** Estazolam. **Panel D**. Sliding-scale insulin. **Panel E.** Zolpidem. **Panel F.** Alprazolam. PIM – potentially inappropriate medication.

**Figure 2:**
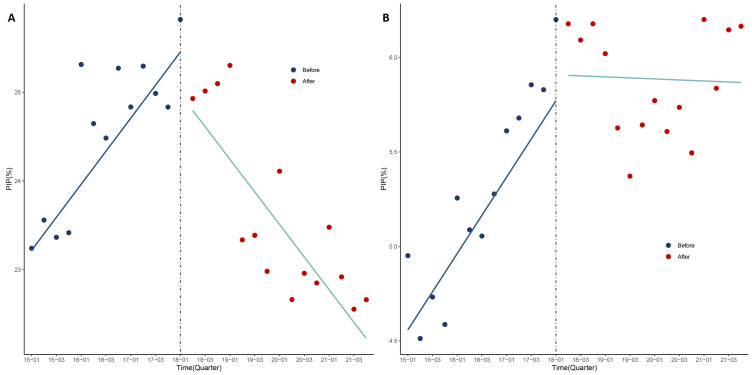
Prevalence of single and multiple PIM use before and after the release of the criteria. **Panel A.** Single PIM. **Panel B.** Multiple PIMs. PIM – potentially inappropriate medication.

Before the release of the criteria, the overall prevalence of PIM showed a statistically significant upward trend. We observed first-order autocorrelation, with the DW statistic being 1.138 pre-adjustment and 1.741 (β = 0.406; 95% CI = 0.341, 0.651; *P* = 0.001) post-adjustment (**Figure 1**, Panel A, **Table 3**). Similarly, the prevalence of single PIM (β = 0.185; 95% CI = 0.110, 0.260; *P* < 0.001; DW = 1.530) and the prevalence of multiple PIMs (β = 0.101; 95% CI = 0.089, 0.161; *P* = 0.001; DW = 2.288) both significantly increased (**Figure 2**, Panels A and B).

**Table 3:** Interrupted time series regression analysis of the prevalence of PIM use

	Intercept		Pre-guideline trajectory		Change associated with guideline release		Post-guideline trajectory	
**Variable**	***β_0_* (95% CI)**	***P*-value**	***β_1_* (95% CI)**	***P*-value**	***β_2_* (95% CI)**	***P*-value**	***β_3_* (95% CI)**	***P*-value**
PIM	25.851 (24.044, 26.573)	<0.001	0.406 (0.341, 0.651)	0.001	−0.125 (−2.635, 0.534)	0.897	−0.607 (−0.881, −0.482)	<0.001
Single PIM	23.067 (22.457, 23.677)	<0.001	0.185 (0.110, 0.260)	<0.001	−0.502 (−1.266, 0.262)	0.188	−0.368 (−0.465, −0.272)	<0.001
Multiple PIMs	4.457 (4.033, 4.624)	<0.001	0.101 (0.089, 0.161)	0.001	0.137 (−0.488, 0.251)	0.540	−0.104 (−0.173, −0.080)	0.019
Clopidogrel	8.299 (7.818, 9.316)	<0.001	0.107 (0.033, 0.217)	0.167	0.152 (−1.625, 0.253)	0.782	−0.342 (−0.463, −0.227)	0.006
Estazolam	3.899 (3.581, 4.135)	<0.001	0.084 (0.056, 0.124)	<0.001	−0.011 (−0.427, 0.268)	0.956	−0.077 (−0.124, −0.037)	0.009
Sliding-scale insulin	3.434 (3.254, 3.615)	<0.001	−0.014 (−0.036, 0.009)	0.219	0.123 (−0.103, 0.349)	0.270	−0.001 (−0.029, 0.028)	0.973
Zolpidem	1.454 (1.280, 1.628)	<0.001	0.054 (0.033, 0.075)	<0.001	0.112 (−0.106, 0.330)	0.300	0.030 (0.002, 0.057)	0.036
Alprazolam	1.499 (1.284, 1.750)	<0.001	0.050 (0.017, 0.074)	0.015	−0.094 (−0.386, 0.197)	0.595	0.001 (−0.026, 0.048)	0.974

CI – confidence interval, PIM – potentially inappropriate medication

There were no significant changes in the overall PIM prevalence, the prevalence of single PIM, or the prevalence of multiple PIMs following the release of the criteria. However, we observed a statistically significant decline in the overall prevalence of PIM (β = −0.607; 95% CI = −0.881, −0.482; *P* < 0.001), the prevalence of single PIM (β = −0.368; 95% CI = −0.465, −0.272; *P* < 0.001), and the prevalence of multiple PIMs (β = −0.104; 95% CI = −0.173, −0.080; *P* = 0.019). These findings suggest that the criteria had a significant long-term impact on reducing the prevalence of PIM, although they did not have an immediate effect.

There was a significant upward trend in the prevalence of estazolam (β = 0.084; 95% CI = 0.056, 0.124; *P* < 0.001), zolpidem (β = 0.054; 95% CI = 0.033, 0.075; *P* < 0.001), and alprazolam (β = 0.050; 95% CI = 0.017, 0.074; *P* = 0.015) (**Figure 1**, Panels B–F, **Table 3**). We found no significant changes in the prevalence for the top five PIMs after the release of the criteria, but did note a decrease in the prevalence of clopidogrel (β = −0.342; 95% CI = −0.463, −0.227; *P* = 0.006; DW = 1.883) and estazolam (β = −0.077; 95% CI = −0.124, −0.037; *P* = 0.009; DW = 1.981). Conversely, the prevalence of zolpidem significantly increased (β = 0.030; 95% CI = 0.002, 0.057; *P* = 0.036; DW = 2.252). The trends for sliding-scale insulin and alprazolam did not significantly change.

## DISCUSSION

To the best of our knowledge, this is the first study to assess the impact of Chinese criteria on PIM use among older outpatients. We included a total of 982 606 patients and found a gradual increase in the prevalence of PIM use, peaking in 2018 before gradually declining. In contrast, studies from Europe and the USA have reported a decreasing trend in recent years [24–27], likely due to the widespread adoption of the STOPP/START and Beers criteria, which have heightened clinician awareness. However, our findings suggest a different trend in China in terms of these two criteria, as PIM use in the country increased before the release of the national criteria. This pattern is similar to findings from Ireland, where PIM use rose from 32.6% in 1997 to 37.3% in 2012 [28].

China released its PIM criteria in 2018 to improve the management of PIM use among older adults and ensure rational drug use. Our results showed that the prevalence of PIM use began to decline with a delay after the release of the criteria, indicating a long-term effect of the criteria on the overall prevalence of PIM use. Considering that no other policies on PIM use were released around 2018, this would suggest that these criteria had an effect in terms of reducing PIM use. Additionally, as clinicians and pharmacists became more familiar with these criteria, it is likely that their application in practice further contributed to this decline. Strengthening the implementation of these criteria in outpatient services and increasing awareness among health care professionals and older adults remains essential, however.

Patterns similar to those we found in our study have been observed in other areas. For instance, one analysis found a decrease in carbanepem use in Shaanxi Province from 2017–20 following the release of related guidelines by the China’s National Health Commission in 2018 [29]. Another study examining the effects of guidelines on key monitored drug use in Xi’an tertiary medical institution from 2014–21 observed a downward trend in both the use and cost of these drugs after the release of the drug list [30]. These results highlight that effective interventions or guidelines can significantly improve rational drug use in hospitals.

The change in the overall prevalence of PIM use after the release of the criteria was not statistically significant, suggesting that the criteria did not have an immediate impact. Research has indicated that the effects of newly introduced criteria are often not immediate [31], as their implementation involves several stages. Initially, stakeholders such as government agencies, health care decision-makers, and clinicians need time to understand the criteria’s content, objectives, and potential impact. Following this, health care institutions and clinicians may need to adapt and adjust, revise clinical workflows, update medication protocols, and conduct staff training. The effectiveness of the criteria then needs to be monitored and evaluated to ensure that the expected outcomes are achieved, a process that can be lengthy and may require ongoing modifications. Finally, the influence of criteria may take time to spread and be fully realised across the health care system [32].

The top five detected drugs included three benzodiazepines, which can be attributed to the high prevalence of insomnia among older adults. Over 80% of patients are prescribed treatment for insomnia, yet most of the prescribed benzodiazepines and sedative-hypnotics are not suitable for older adults [33]. As the body's organs and tissues gradually age, older adults become more sensitive to medications. Despite the association of benzodiazepines with increased risks of cognitive impairment, falls, and fractures, they remain commonly prescribed for older adults [34]. The relevant criteria recommend that the treatment of insomnia in older adults should initially involve nonpharmacological therapies, including stimulus control, sleep restriction, relaxation training, and cognitive behavioural therapy [35,36]. A review reported that cognitive behavioural therapy is considered the first-line treatment for insomnia, with benzodiazepines being discouraged for older adults, especially for long-term use [37]. Z-drugs are considered the first-line treatment for insomnia patients because of their pharmacokinetic properties and safety profile. However, research evidence suggests that they carry similar risks of ADRs as benzodiazepines, such as impaired physical balance and increased risk of falls in older adults [38]. A comparative study of zolpidem and benzodiazepines revealed that zolpidem is associated with a greater risk of fracture than alprazolam and lorazepam [39].

After the release of the criteria, the prevalence of PIM use associated with estazolam decreased, whereas the prevalence of PIM use associated with zolpidem increased. Compared with benzodiazepine sedatives such as alprazolam and estazolam, zolpidem has a similar hypnotic effect, but a shorter half-life, causing less disruption to normal sleep architecture. It is considered safer than benzodiazepines because it has fewer daytime sedative effects and other adverse reactions, which contributes to its widespread use [40]. We theorise that clinicians substituted benzodiazepines with zolpidem due to misconceptions about safety. Benzodiazepines such as alprazolam and estazolam are classified as the top five PIMs in the Chinese criteria, as well as in the Beers and STOPP/START criteria, and should be avoided (data not yet published). The prevalence of PIM use associated with clopidogrel also decreased after the release of the criteria, likely because clinicians are increasingly opting for newer drugs in the same class, such as ticagrelor. Research indicates that, compared with clopidogrel, ticagrelor results in fewer ischemic events in older adults without increasing the risk of bleeding [41].

When reducing the use of PIM, it is crucial not only to consider medical evidence and the patient’s disease status, but also to effectively manage and guide the patient's preferences. Strengthening doctor-patient communication, increasing patient awareness of drug risks, and promoting patient participation in treatment decisions are key strategies to reduce PIM use. Reducing PIM use not only enhances drug safety, decreases ADRs, and improves overall patient health, but also optimises treatment options and lowers the risk of hospitalisation and readmissions. These benefits collectively contribute to better clinical outcomes and an improved quality of life for older patients.

This study has several limitations. First, although the data were collected from multiple hospitals across six geographic regions in China, they were limited to older outpatients in secondary and tertiary hospitals. Consequently, the findings may not fully reflect PIM use among older patients in primary health care settings. Second, due to the lack of follow-up data, we were unable to analyse the adverse health outcomes associated with PIM use or assess the impact of the Chinese criteria on individual clinical outcomes, such as ADRs or all-cause mortality. Third, while interrupted time series analysis can identify trends, it does not establish direct causality. External factors may have influenced our results. Therefore, further research is needed to clarify the immediate effects of the Chinese criteria on PIM use.

## CONCLUSIONS

Our findings suggest that the release of the criteria influenced the overall prevalence of PIM use among older outpatients, though no immediate effects were observed. However, the criteria did not impact the short-term or long-term prevalence of zolpidem, sliding-scale insulin, or alprazolam use. Therefore, further efforts are needed to strengthen the management of these specific PIMs.

## Additional material


Online Supplementary Document
