# Supplementary material for: Impact of Chinese criteria on potentially inappropriate medication use in China
Source: J Glob Health. 2025 Feb 7;15:04063. doi: 10.7189/jogh.15.04063 (PMC11801653; doi:10.7189/jogh.15.04063)
Supplement: Online Supplementary Document [file jogh-15-04063-s001.pdf]

Supplement to: Tian F, Chen Z, Zhang Y, Feng Q, Chen X. Impact of Chinese criteria on potentially inappropriate medication use in China. J Glob Health. 2025;15:04063.

Impact of Chinese criteria on potentially inappropriate medication use in China

SUPPLEMENTARY APPENDIX

Contents:

Appendix 1. Basic information of the hospitals included in the study
Appendix 2. Basic characteristics of patients for every year
Appendix 3. Basic characteristics of study PIM use

Appendix 1. Basic information of the hospitals included in the study

City	Hospital code	No	
		**2nd**	**3nd**
Beijing	211, 214, 301, 302, 304, 310, 313, 318, 319	2	7
Chengdu	211, 212, 213, 305, 306, 307, 308, 310, 312	3	6
Guangzhou	301, 302, 305, 306, 309, 314	0	6
Shanghai	208, 218, 220, 223, 227, 302, 305, 306, 309, 310, 311, 312, 314, 321, 322, 326, 328	5	12
Shenyang	301, 303, 304, 305, 306, 308, 309, 310, 311, 312, 313	0	11
Zhengzhou	207, 304, 306, 308, 313, 314, 316	1	6
Total		11	48

Appendix 2. Basic characteristics of patients for every year

Characteristic	2015	2016	2017	2018	2019	2020	2021	Total
City								
Beijing	31,582 (27.22)	32,519 (23.45)	31,725 (22.15)	34,984 (22.70)	34,696 (20.97)	19,407 (14.58)	18,429 (13.97)	203,342 (20.69)
Chengdu	10,216 (8.80)	12,964 (9.35)	10,978 (7.66)	12,928 (8.39)	13,313 (8.05)	11,998 (9.01)	8,543 (6.48)	80,940 (8.24)
Guangzhou	10,507 (9.05)	25,814 (18.62)	24,179 (16.88)	23,410 (15.19)	21,986 (13.29)	16,678 (12.53)	17,737 (13.45)	140,311 (14.28)
Shanghai	45,219 (38.97)	46,415 (33.47)	48,560 (33.90)	46,845 (30.39)	51,354 (31.03)	41,660 (31.29)	44,176 (33.49)	324,229 (33.00)
Shenyang	16,903 (14.57)	19,135 (13.80)	25,271 (17.64)	32,839 (21.30)	40,722 (24.61)	40,367 (30.32)	39,115 (29.66)	214,352 (21.81)
Zhengzhou	1,610 (1.39)	1,822 (1.31)	2,519 (1.76)	3,135 (2.03)	3,410 (2.06)	3,041 (2.28)	3,894 (2.95)	19,431 (1.98)
Hospital level								
2nd	10,435 (8.99)	11,687 (8.43)	15,527 (10.84)	13,564 (8.80)	12,180 (7.36)	9,808 (7.37)	10,314 (7.82)	83,515 (8.50)
3nd	105,602 (91.01)	126,982 (91.57)	127,705 (89.16)	140,577 (91.20)	153,301 (92.64)	123,343 (92.63)	121,580 (92.18)	899,090 (91.50)
Sex								
Male	74,466 (64.17)	89,131 (64.28)	90,863 (63.44)	98,663 (64.01)	105,690 (63.87)	85,147 (63.95)	83,369 (63.21)	627,329 (63.84)
Female	41,571 (35.83)	49,538 (35.72)	52,369 (36.56)	55,478 (35.99)	59,791 (36.13)	48,004 (36.05)	48,525 (36.79)	355,276 (36.16)
Age group, years								
65-79	49,231 (42.43)	58,638 (42.29)	59,823 (41.77)	65,406 (42.43)	72,837 (44.02)	61,544 (46.22)	63,893 (48.44)	431,372 (43.90)
≥80	66,806 (57.57)	80,031 (57.71)	83,409 (58.23)	88,735 (57.57)	92,644 (55.98)	71,607 (53.78)	68,001 (51.57)	551,233 (56.10)
Total	116,037	138,669	143,232	154,141	165,481	133,151	131,894	982,605

Appendix 3. Basic characteristics of study PIM use

**Characteristic**	**2015**	**2016**	**2017**	**2018**	**2019**	**2020**	**2021**	**Total**
Patients of PIMs								
Single PIM use	27,143 (74.06)	34,581 (82.82)	35,782 (81.31)	38,892 (80.37)	39,254 (80.73)	30,817 (80.38)	30,219 (79.01)	236,688 (81.07)
Multiple PIM use	5,456 (16.74)	7,171 (17.18)	8,227 (18.69)	9,802 (19.63)	9,371 (19.27)	7,523 (19.62)	8,026 (20.99)	55,276 (18.93)
City								
Beijing	11,267 (34.56)	12,029 (28.81)	11,426 (25.96)	12,454 (25.73)	11,590 (23.84)	6,625 (17.28)	6,637 (17.35)	72,028 (24.67)
Chengdu	3,692 (11.33)	4,537 (10.87)	4,273 (9.71)	4,905 (10.14)	4,550 (9.36)	4,216 (11.00)	3,056 (7.99)	29,229 (10.01)
Guangzhou	2,646 (8.12)	8,491 (20.34)	8,993 (20.43)	9,857 (20.37)	9,687 (19.92)	7,451 (19.43)	7,400 (19.35)	54,525 (18.68)
Shanghai	10,592 (32.49)	11,308 (27.08)	12,139 (27.58)	11,653 (24.08)	12,552 (25.81)	10,871 (28.35)	11,084 (28.98)	80,199 (27.47)
Shenyang	3,775 (11.58)	4,660 (11.16)	6,118 (13.90)	8,134 (16.81)	8,825 (18.15)	7,950 (20.74)	8,449 (22.09)	47,911 (16.41)
Zhengzhou	627 (1.92)	727 (1.74)	1,060 (2.41)	1,391 (2.87)	1,421 (2.92)	1,227 (3.20)	1,619 (4.23)	8,072 (2.76)
Hospital level								
2nd	2,984 (9.15)	3,598 (8.62)	4,510 (10.25)	3,631 (7.50)	3,117 (6.41)	2,813 (7.34)	3,013 (7.88)	23,666 (8.11)
3nd	29,615 (90.85)	38,154 (91.38)	39,499 (89.75)	44,763 (92.50)	45,508 (93.59)	35,527 (92.66)	35,232 (92.12)	268,298 (91.89)
Sex								
Male	21,884 (67.13)	28,101 (67.30)	29,210 (66.37)	31,760 (65.63)	31,522 (64.83)	24,107 (62.88)	24,035 (62.84)	190,619 (65.29)
Female	10,715 (32.87)	13,651 (32.70)	14,799 (33.63)	16,634 (34.37)	17,103 (35.17)	14,233 (37.12)	14,210 (37.16)	101,345 (34.71)
Age group, years								
65-79	12,138 (37.23)	15,512 (37.15)	15,999 (36.35)	18,194 (37.60)	18,798 (38.66)	15,730 (41.03)	16,100 (42.10)	112,471 (38.52)
≥80	20,461 (62.77)	26,240 (62.85)	28,010 (63.65)	30,200 (62.40)	29,827 (61.34)	22,610 (58.97)	22,145 (57.90)	179,493 (61.48)
Total	32,599	41,752	44,009	48,394	48,625	38,340	38,245	291,964
